# Epigenome targeting by probiotic metabolites

**DOI:** 10.1186/1757-4749-2-24

**Published:** 2010-12-21

**Authors:** Paul V Licciardi, Sook-San Wong, Mimi LK Tang, Tom C Karagiannis

**Affiliations:** 1 Allergy and Immune Disorders, Murdoch Children's Research Institute, Melbourne, Australia; 2 Allergy and Immunology, Royal Children's Hospital, Melbourne, Australia; 3 Department of Paediatrics, University of Melbourne, Melbourne, Australia; 4 Epigenomic Medicine, Baker IDI Heart and Diabetes Institute, Melbourne, Australia; 5 Department of Pathology, University of Melbourne, Melbourne, Australia

## Abstract

**Background:**

The intestinal microbiota plays an important role in immune development and homeostasis. A disturbed microbiota during early infancy is associated with an increased risk of developing inflammatory and allergic diseases later in life. The mechanisms underlying these effects are poorly understood but are likely to involve alterations in microbial production of fermentation-derived metabolites, which have potent immune modulating properties and are required for maintenance of healthy mucosal immune responses. Probiotics are beneficial bacteria that have the capacity to alter the composition of bacterial species in the intestine that can in turn influence the production of fermentation-derived metabolites. Principal among these metabolites are the short-chain fatty acids butyrate and acetate that have potent anti-inflammatory activities important in regulating immune function at the intestinal mucosal surface. Therefore strategies aimed at restoring the microbiota profile may be effective in the prevention or treatment of allergic and inflammatory diseases.

**Presentation of the hypothesis:**

Probiotic bacteria have diverse effects including altering microbiota composition, regulating epithelial cell barrier function and modulating of immune responses. The precise molecular mechanisms mediating these probiotic effects are not well understood. Short-chain fatty acids such as butyrate are a class of histone deacetylase inhibitors important in the epigenetic control of host cell responses. It is hypothesized that the biological function of probiotics may be a result of epigenetic modifications that may explain the wide range of effects observed. Studies delineating the effects of probiotics on short-chain fatty acid production and the epigenetic actions of short-chain fatty acids will assist in understanding the association between microbiota and allergic or autoimmune disorders.

**Testing the hypothesis:**

We propose that treatment with specific probiotic bacteria under *in vivo *conditions would offer the ideal conditions to examine the microbiological, immunological and epigenetic mechanisms of action. Advances in epigenetic technology now allow investigators to better understand the complex biological properties of probiotics and their metabolites.

**Implications of the hypothesis:**

Determining the precise mechanisms of probiotic action will lead to more specific and efficacious therapeutic strategies in the prevention or treatment of chronic inflammatory conditions.

## Background

The intestinal microbiota plays a critical role in the establishment and maintenance of healthy immune responses. Delayed colonisation of the infant gut with commensal bacteria or alterations in the microbiota profile are suggested to be strong risk factors for the development of immune-mediated chronic disorders such as allergic and autoimmune diseases. Mice raised in a germ- free environment fail to develop oral tolerance and have persistent Th2-dependent immune responses [1]. This immune deviation can be corrected by reconstituting the microbiota with a single bacteria species, but only if this occurs during the neonatal period. Similarly, infants with allergic disease have reduced numbers of beneficial *Bifidobacteria *species and increased numbers of pathogenic *Clostridia *and *Staphylococci *compared to non-allergic infants [2-4]. Moreover, these changes occur prior to the onset of allergy, suggesting a causal relationship between microbiota and healthy immune responses. These observations have led to the idea that probiotic bacteria - which have the potential to restore the intestinal microbiota balance - may be effective in preventing the development of chronic immune-mediated diseases.

Probiotics mediate their activity by a variety of mechanisms including altering the microbiota composition, maintaining epithelial barrier function and modulating mucosal and systemic immune responses [5]. Importantly, the effects of probiotic bacteria are species and strain-specific, so it is imperative to select probiotic bacteria with specific activities based upon known *in vitro *or *in vivo *effects that are relevant to the clinical context they will be applied to. *Lactobacillus *and *Bifidobacteria *species have been studied in greatest detail and are the most commonly used in research studies [6]. In particular, significant effort has been made in the examination of probiotic therapy for the prevention of allergic disease. Several clinical trials have demonstrated that a combined prenatal/early postnatal treatment has the greatest impact on reducing allergy symptoms, indicating the importance of early life interventions [7-9]. Prenatal supplementation with the probiotic *Lactobacillus rhamnosus *GG (LGG) was found to reduce the development of atopic dermatitis in high-risk infants by 2 years of age [7]. However, subsequent studies using the same or different probiotics have not confirmed these results, suggesting that there may be intrinsic differences between the probiotic strains used and study populations, reflecting the complexity of these studies [10].

Treatment with probiotics induces a variety of immunological effects on epithelial function, dendritic cells, Treg and T-helper responses. Examples of these effects include 1) enhanced epithelial barrier function via interactions with Toll-like receptors (TLRs) and modulation of epithelial cell signal transduction pathways that regulate cytokine production to promote anti-inflammatory responses [11-13]; 2) induction of tolerogenic DCs producing low IFNγ and elevated IL-10 [14]; 3) induction of Treg activity associated with increased TGF-β and IL-10 secretion by PBMCs [15,16]; 4) modulation of T helper responses [17]; 5) stimulation of IgA responses to oral and parenteral vaccines [18-20] and 6) modulation of immune factors within breast milk such as TGF-β, soluble CD14 and total IgA [14,21]. Genetic analysis of probiotic effects following treatment of healthy adults with *L. casei *and *L. rhamnosus *revealed upregulation of several key mucosal immune response genes encoding IFN-γ production and TLR3/9 expression while *L. acidophilus *was found to upregulate immunoregulatory genes including IL-17B, IRAK2 as well as several chemokines and cellular adhesion molecules in duodenal biopsies using a novel transcriptome analysis [22].

An important role for commensal (including probiotic) bacteria is the fermentation of dietary compounds leading to production of short-chain fatty acid (SCFA) metabolites. Several SCFAs are known to be produced such as butyrate, acetate and propionate. Levels of SCFA have been demonstrated to be reduced in inflammatory diseases, possibly as a result of an altered gut microbiota. These SCFAs have an important protective function in the intestine and exert anti-inflammatory effects in a number of animal models. Intestinal epithelial cells of germ-free mice had reduced SCFA receptor expression which was normalized following reconstitution with normal gut bacteria [23]. The SCFA, butyrate, also has an important role as an energy source for the mucosa in predominantly anaerobic environments such as the gastrointestinal tract as well as having other critical biological functions such as immune regulation [24]. Moreover, butyrate improves epithelial barrier integrity by modulating the expression of certain tight junction proteins such as cingulin, ZO proteins and occludin [25,26]. These combined effects promote establishment of a healthy microbiota and the development of a healthy gastrointestinal lymphoid tissue. Studies involving SCFA treatment using butyrate and acetate have yielded promising results, with amelioration of inflammatory lesions in mouse models of allergic airways disease and colitis [27]. This has led to the idea that probiotic supplementation could restore SCFA levels by modulating the microbial environment, representing a novel therapeutic or preventative strategy for chronic inflammatory diseases.

Probiotic metabolites such as butyrate are therefore an important class of therapeutic compounds. These SCFAs are also a well-known class of epigenetic drugs known as histone deacetylase inhibitors (HDACi) that have a central role as anti-cancer agents with strong anti-proliferative effects on tumour cells [28,29]. This activity of SCFAs may explain the anti-inflammatory effects observed for probiotic bacteria. Intriguingly, the principal HDACi compound is butyric acid (sodium butyrate) which inhibits most HDAC enzymes except class III and class II HDACs 6 and 10 [30]. Butyrate is one of the most potent HDACi in human colon cancer cell lines suggesting an integral role as anti-inflammatory derivatives of microbial fermentation in the colon [24]. Studies using fecal fermentation supernatants were found to be rich in butyrate and exhibited strong HDAC inhibitory properties in several colon cancer cell lines [31]. The HDACi activity of butyrate and propionate was associated with blockade of DC development primarily through the Na^+ ^coupled monocarboxylate transporter Slc5a8 [32].

Given the role of bacterial species in the production of SCFAs, probiotics may be considered as an alternative approach for the prevention or treatment of chronic inflammatory diseases. Evidence in support of this effect by probiotics is limited. The butyric acid-producing anaerobic bacterium, *Faecalibacterium prausnitzii*, is a novel probiotic used for the treatment of inflammatory bowel disease (IBD) [33]. The levels of this bacterium is lower in IBD and treatment of mice with *F. prausnitzii *led to a shift in microbiota composition, reduced inflammatory cytokine levels such as IL-2, increased the anti-inflammatory cytokine IL-10 and reduced colitis and mortality, suggesting that butyrate and other related metabolites may be critical in host protection [34]. The anti-inflammatory effects of SCFA-producing probiotic bacterium was further illustrated with *Propionibacterium freudenreichii*, which was found to shift the extracellular pH from 7.5 to 5.5 in the colon and was related to levels of the SCFAs, acetate and propionate [35]. It is possible that probiotic-derived SCFA metabolites such as butyrate produce HDACi effects that have multiple downstream effector functions on many target cells, including those of the immune system. This offers a promising intervention approach for the treatment of inflammatory conditions such as allergy and gastrointestinal disease. The exact mechanism(s) of action for probiotic bacteria have yet to be fully understood; therefore the epigenomic-modifying capacity of probiotics will be important in understanding how they mediate their health-promoting effects.

## Presentation of the hypothesis

Probiotics mediate pleiotropic effects however the precise mechanism(s) have yet to be fully elucidated. The 'epigenome targeting' hypothesis contends that the activity of probiotics and their metabolites are associated with chromatin remodeling as a function of intrinsic histone deacetylase inhibition. Modulation of gene transcription is an essential component of many biological processes. The onset of pathological conditions such as cancer and chronic inflammation often result from aberrant gene transcription. Integral to this process are epigenetic factors involving two critical enzymes, histone acetylases (HATs) and histone deacetylases (HDACs). These produce post-translational modifications of histone proteins and result in changes to chromatin structure and function [36,37]. While HATs serve to acetylate histones, conferring a 'relaxed' chromatin structure that allows transcriptional activation, HDACs has the opposite effect. HDAC enzymes repress transcription through tightening of the chromatin structure, excluding accessibility of transcription factors and other regulatory proteins to DNA and therefore the ability to influence gene expression [38].

The recent advances in understanding epigenetic processes in the maintenance of health allows us to investigate more precisely the mechanisms of action of novel therapies such as probiotics in defined clinical contexts. Critical transcriptional regulators of inflammation such as NF-κB and Foxp3 can now be studied closely to determine the impact of dietary/probiotic supplementation. The evidence presented above and our hypothesis provide for the first time a direct link between the anti-inflammatory and immunoregulatory properties observed for probiotics in terms of Treg induction, DC maturation and cytokine/chemokine secretion and the molecular events involved at the epigenetic level. We believe that the biological activity of probiotics are mediated through complex epigenetic changes that regulate the activation status of key transcription factors involved in host immunity.

## Testing the hypothesis

Testing this hypothesis should be undertaken in an *in vivo *system such as the mouse, or preferably in humans using a randomized, double-blind, placebo-controlled study design. Probiotic bacteria or placebo should be given prenatally and/or post-natally to infants at high-risk of developing allergic disease. Clinical effects of the probiotic should be assessed by doctor-diagnosed allergy using defined criteria, skin prick test positivity, and SCORAD analysis of eczema severity. Immunological outcomes can be assessed by enumerating Treg and DC populations and the profile of cytokine secretion by cultured cord and peripheral blood mononuclear cells in response to SCFA metabolites. Levels of SCFA can be measured in stool or blood. Correlation between immunological and epigenetic changes could be assessed in the first instance by simple immunoblot analysis for histone hyperacetylation as well as real-time PCR for specific immune gene regulation in relevant cells. Genome-wide sequencing techniques such as mRNA-Seq (gene expression) and ChIP-Seq (specific epigenetic modifications including acetylation) will further our understanding of the critical biological functions of probiotics.

## Implications of the hypothesis

Interest in probiotics has intensified in recent years due to their potential health benefits and as treatments for a wide range of inflammatory and immunological conditions. Probiotic bacteria have been demonstrated to modulate a variety of microbiological and immunological parameters although the precise mechanisms for these effects are yet to be fully elucidated. The ability of probiotics to alter intestinal microbiota composition and diversity suggest an important role for SCFAs such as butyrate. The demonstration of beneficial probiotic effects through as yet unidentified epigenetic mechanisms will have significant implications in the prevention and/or treatment of allergy and other chronic inflammatory diseases.

## Competing interests

PVL, SSW and TCK declare that they have no competing interests. MLKT is a member of the medical advisory board of Nestle Nutrition Institute and has delivered speaker presentations for Wyeth/Pfizer.

## Authors' contributions

PVL conceived and wrote the paper, SSW contributed to the manuscript, MLKT and TCK contributed to and critically appraised the manuscript. All authors read and approved the final manuscript.
